# Necessity of organized low-dose computed tomography screening for lung cancer: From epidemiologic comparisons between China and the Western nations

**DOI:** 10.18632/oncotarget.12400

**Published:** 2016-10-01

**Authors:** Hong-Feng Gou, Yang Liu, Tian-Xia Yang, Cheng Zhou, Xin-Zu Chen

**Affiliations:** ^1^ Department of Medical Oncology, Cancer Center, State Key Laboratory of Biotherapy, West China Hospital, Sichuan University, Chengdu, China; ^2^ West China School of Public Health, Sichuan University, Chengdu, China; ^3^ Department of Nuclear Medicine, West China Hospital, Sichuan University, Chengdu, China; ^4^ Translational Radiation Oncology, National Center for Tumor Diseases (NCT), German Cancer Research Center (DKFZ), Heidelberg, Germany; ^5^ Heidelberg Ion Therapy Center (HIT), Department of Radiation Oncology, University Heidelberg Medical School, Heidelberg, Germany; ^6^ German Consortium for Translational Cancer Research (DKTK), German Cancer Research Center (DKFZ), Heidelberg, Germany; ^7^ Department of Gastrointestinal Surgery, West China Hospital, Sichuan University, Chengdu, China

**Keywords:** lung cancer, screening, low-dose computed tomography, epidemiology, mortality

## Abstract

**Objectives:**

To compare the proportion of stage I lung cancer and population mortality in China to those in U.S. and Europe where lung cancer screening by low-dose computed tomography (LDCT) has been already well practiced.

**Methods:**

The proportions of stage I lung cancer in LDCT screening population in U.S. and Europe were retrieved from NLST and NELSON trials. The general proportion of stage I lung cancer in China was retrieved from a rapid meta-analysis, based on a literature search in the China National Knowledge Infrastructure database. The lung cancer mortality and prevalence of China, U.S. and Europe was retrieved from Globocan 2012 fact sheet. Mortality-to-prevalence ratio (MPR) was applied to compare the population survival outcome of lung cancer.

**Results:**

The estimated proportion of stage I lung cancer in China is merely 20.8% among hospital-based cross-sectional population, with relative ratios (RRs) being 2.40 (95% CI 2.18–2.65) and 2.98 (95% CI 2.62–3.38) compared by LDCT-screening population in U.S. and Europe trials, respectively. MPR of lung cancer is as high as 58.9% in China, with RRs being 0.46 (95% CI 0.31–0.67) and 0.58 (95% CI 0.39–0.85) compared by U.S. and Europe, respectively.

**Conclusions:**

By the epidemiological inference, the LDCT mass screening might be associated with increasing stage I lung cancer and therefore improving population survival outcome. How to translate the experiences of lung cancer screening by LDCT from developed counties to China in a cost-effective manner needs to be further investigated.

## INTRODUCTION

The stage of the lung cancer is an important prognostic factor, and therefore the opportunity for curative treatment and long-term survival outcome declines with more advanced diseases [1, 2]. By now, there is still no definitive biomarker or genetic factor for prediction or early diagnosis of lung cancer [3]. Low-dose computed tomography (LDCT) for lung cancer screening is recommended as a promising approach to reducing mortality from lung cancer by NLST and NELSON trials in U.S. and Europe respectively [4, 5]. These trials found that LDCT screening was able to significantly increase the proportion of stage I diseases among detected lung cancer. Consequently, the overall survival of LDCT screening population may be obviously improved compared to population with conventional chest X-ray screening or without screening. Clinical practice guidelines also recommended the LDCT screening in the high-risk subpopulation [6].

In China, lung cancer is always the leading cause of cancer death [7]. However, LDCT has not been applied as a preferred screening approach to lung cancer in China. The necessity of LDCT for lung cancer screening in mass health examination needs to be underlined in China. Therefore, to provide epidemiological evidence, we compare the proportion of stage I lung cancer and population mortality in China to those in U.S. and Europe, where LDCT screening for lung cancer has been already well practiced.

## RESULTS

Meta-analysis based on 2,585 patients from 8 eligible studies demonstrates that the general proportion of stage I lung cancer in China is merely 20.8% (Table 1). The patient characteristics are shown in Table 2. Comparing the proportions in LDCT-screening populations from NLST and NELSON trials to that in China, the RRs for proportions of U.S. or Europe to China are 2.40 (95% confidence interval [CI] 2.18–2.65) and 2.98 (95% CI 2.62–3.38), respectively (p<0.0001) (Table 3). On the other hand, despite of a lower prevalence, the MPR of lung cancer is as high as 58.9% (95% CI 57.1%–60.6%) in China, compared to only 26.9% (95% CI 26.1%–27.7%) and 33.9% (95% CI 32.6%–35.3%) in U.S. and Europe, respectively (Table 3). The RRs for MPRs of U.S. or Europe to China are 0.46 (95% CI 0.31–0.67) and 0.58 (95% CI 0.39–0.85), respectively (*p*<0.005).

**Table 1:** A pooling estimate on the proportion of stage I lung cancer among Chinese population during 2012–2014

Study	Stage I (n)	Sample (N)	Proportion (95% CI)	COPD status
Chen R, 2014	165	816	20.2% (17.6%–23.1%)	Any
Lou J, 2014	294	1329	22.1% (20.0%–24.4%)	Any
Li LX, 2013	0	110	0.5% (0.0%–6.8%)	Prevalent
Sun HB, 2013	24	100	24.0% (16.6%–33.3%)	Prevalent
Yi ZM, 2013	0	42	1.2% (0.1%–16.0%)	Prevalent
Bo XX, 2012	2	48	4.2% (3.4%–5.0%)	Prevalent
Lin XF, 2012	44	105	41.9% (32.9%–51.5%)	Prevalent
Wang Y, 2012	6	35	17.1% (7.9%–33.3%)	Prevalent
Pooling estimate (random effect)*	535	2585	20.8% (15.3%-27.5%)	

Abbreviations: CI, confidence interval; COPD, chronic obstructive pulmonary diseases.

* Heterogeneity test: I-square=84.1%, *p*<0.001.

**Table 2:** The patient characteristics of included Chinese hospital-based studies

Study	Sample	Male % (95 CI)	Age (yrs) mean±SD (range)	Current or former smoker % (95 CI)	SCLC % (95 CI)	SCC % (95 CI)
Chen R, 2014	816	66.3 (66.2-66.4)	56.8±2.6 (24-75)	67.2 (67.0-67.3)	47.4 (47.3-47.5)	39.3 (39.2-39.5)
Lou J, 2014	1329	74.6 (74.6-74.7)	58 (26-81)	/	26.5 (26.4-26.6)	53.1 (53.0-53.2)
Li LX, 2013	110	80.0 (79.3-80.7)	58.9±10.8 (40-85)	92.7 (92.3-93.2)	10.9 (10.4-11.5)	37.3 (36.4-38.1)
Sun HB, 2013	100	71.0 (70.1-71.9)	69 (56-82)	82.0 (81.2-82.8)	11.0 (10.4-11.6)	46.0 (45.0-47.0)
Yi ZM, 2013	42	76.2 (74.2-78.2)	64.5 (45-81)	85.7 (84.1-87.3)	14.3 (12.7-15.9)	66.7 (64.5-68.9)
Bo XX, 2012	48	91.7 (90.5-92.8)	73.6 (56-89)	91.7 (90.5-92.8)	4.2 (3.4-5.0)	47.9 (45.9-50.0)
Lin XF, 2012	105	88.6 (88.0-89.2)	65.0±8.5	81.9 (81.2-82.6)	9.5 (9.0-10.1)	49.5 (48.6-50.5)
Wang Y, 2012	35	77.1 (74.8-79.5)	63.7±6.7 (54-76)	88.6 (86.8-90.4)	20.0 (17.8-22.2)	45.7 (42.9-48.5)

Abbreviations: CI, confidence interval; SCC, squamous cell carcinoma; SCLC, small cell lung cancer; SD, standard deviation.

**Table 3:** Stage I proportion and population mortality of lung cancer in China different from U.S. and Europe

Outcome	China	U.S.	Europe
Stage I lung cancer			
Proportion, % (95% CI) †	20.8 (15.3–27.5)	50.0 (49.9–50.1)	61.9 (61.5–62.3)
RR of proportion (95% CI)	Reference	2.40 (2.18–2.65)	2.98 (2.62–3.38)
*p* value (2-sided)	—	<0.0001	<0.0001
Population mortality index			
Mortality ‡	32.5	28.6	24.0
Prevalence ‡	55.2	106.4	70.7
MPR, % (95% CI)	58.9 (57.1–60.6)	26.9 (26.1–27.7)	33.9 (32.6–35.3)
RR of MPR (95% CI)	Reference	0.46 (0.31–0.67)	0.58 (0.39–0.85)
*p* value (2-sided)	—	0.0001	0.0041

Abbreviations: CI, confidence interval; MPR, mortality-to-prevalence ratio; RR, relative ratio.

† General proportion of stage I lung cancer in China was retrieved from aforementioned meta-analysis based on Chinese hospital-based cross-sectional studies, while those for U.S. and Europe were based on the LDCT-screening population from NSLT and NELSON trials respectively.^1,2^

‡ Epidemiological data (per 100,000 persons) were retrieved from Globocan 2012 fact sheet.^3^

## DISCUSSION

The significantly higher MPR in China indicates the population survival outcome of lung cancer is apparently worse than that in U.S. and Europe. This unfavorable situation in China may attribute to the lower proportion of stage I disease among detected lung cancers, compared to those LDCT-screening populations. These epidemiological findings imply the disadvantage in detecting early lung cancer in China is largely owing to default of nationwide organized screening programme for lung cancer, especially via LDCT mass screening.

**Figure 1:**
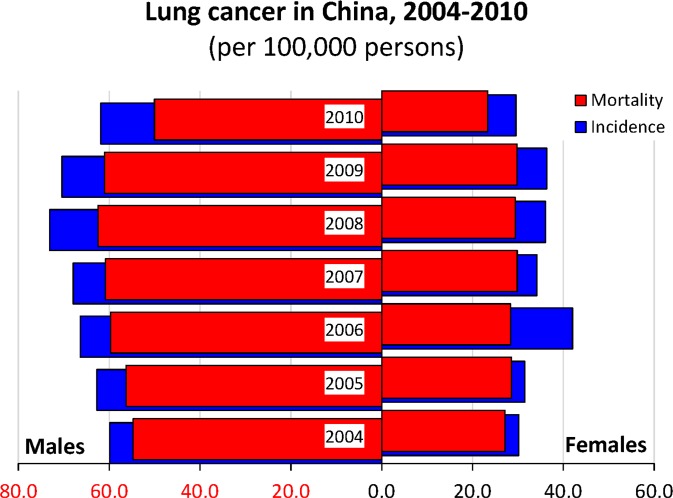
The crude incidence and mortality of lung cancer in mainland China, retrieved from annual reports of National Central Cancer Registry, China Both the incidence and mortality tended to decrease till 2010.

**Figure 2:**
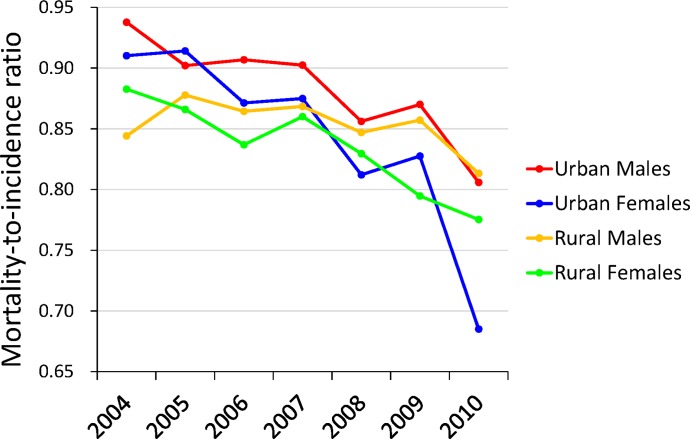
The curves of mortality-to-incidence ratios in mainland China The ratios appeared to decline among any sub-population, especially obviously for urban females. However, till 2010, the ratios were still higher than 0.65 and 0.75 among urban and rural females respectively, while they were always higher than 0.80 among males.

Factually, in 2003, Ministry of Health (MOH), China has issued governmental outlines of China Cancer Prevention and Control Program (2004–2010) [8, 9]. Lung cancer has been defined as one out of eight key malignancies requiring intensive prevention and control in China. The data between 2004 and 2010 reported by the National Cancer Registry, China [10–17] show the incidence and mortality of lung cancer tended to decline to certain extent (Figure 1), especially in the curves of mortality-to-incidence ratios (Figure 2). However, current screening and surveillance programmes for lung cancer are still implemented with loose organization in few major cities in China. Despite of previous efforts of cancer control, our findings demonstrate the population survival outcome of lung cancer is still poor, compared to western developed countries. It implies that it is necessary to find an efficient screening approach to increasing the proportion of early lung cancer and subsequently improving the population survival outcome of lung cancer.

Commonly, the health check-up examination for lung cancer in China is based on chest X-ray for many years. According to NLST trial, LDCT shows better performance for detecting early-stage lung cancer and is associated with decreased mortality from lung cancer [4]. By now, many Chinese hospitals attempt to practice LDCT for lung cancer screening during health check-up instead of traditional chest X-ray test. In the study of Zhang et al. [18], the detection rate of suspicious lesions (at least 1 non-calcified nodule with a diameter ≥4 mm) in lung was 9.9% among 2,251 asymptomatic participants. In the study of Tang et al. [19], the detection rate of lung cancer was 0.6% among 4,690 asymptomatic participants (≥40 years old) from a medical institute in Beijing, and promisingly 76.0% of the detected lung cancers were stage I. In a retrospective study on surgical patients, Lai, et al. found the proportion of stage I lung cancer detected by LDCT was 14% higher than that by chest X-ray examination [20]. In China, initial experiences of screening lung cancer through LDCT were few, but those studies were able to repeat the capacity of increasing the proportion of stage I diseases among detected lung cancers.

Additionally, the healthcare insurance system has already been much advanced than before in mainland China, but the healthcare expenditure planned in the field of cancer screening and surveillance is still limited till now. First of all, the traditional chest radiography (with or without sputum cytology) has been denied as screening techniques for lung cancer [21, 22]. RCTs in the 1960s and 1970s found that chest X-ray screening did not reduce the mortality for high-risk individuals [23]. In contrast, current strong evidence showed that the LDCT mass screening can reduce lung cancer related and all-cause mortality [24]. Actually, it is obviously difficult to establish a national whole-covering lung cancer screening programme based on LDCT at this moment in mainland China. On the other hand, extra expenditure in cancer screening and surveillance might obtain payoffs by preventing healthcare expenditure from adjuvant and palliative treatment for locally advanced and metastatic lung cancer patients, as well as controlling premature death from lung cancer and related disability-adjusted life year. Therefore, how to design a cost-effective screening programme for lung cancer based on LDCT must be a big question for public health administrations in mainland China [22]. Determination of high-risk population for screening and proper interval for surveillance would the two most important problems at the beginning of initial practice of LDCT mass screening among Chinese population. The access to the LDCT screening is also imbalanced between urban and rural areas [20, 25]. How to reduce the urban-rural imbalance of medical resources and healthcare insurance is another issue in designing a cost-effecitve screening program. Due to the uncertainty of feasibility for population-based LDCT screening in China, a demonstration program of lung cancer screening was initiated in the 2010 [26]. High-risk individuals were enrolled in various Chinese centers, which aimed to provide opportunities to explore the feasibility of LDCT lung cancer screening in the Chinese setting [26].

The harm of LDCT massing screening also need consider with caution. Especially, radiation exposure, high false-positive rates, and the potential for overdiagnosis are regarded as major issues in the practice [27]. According to a systematic review, around 20% of screenees had positive findings indicating subsequent follow-up, but just 1% had lung cancer finally [28]. Currently, there is inadequate evidence to support the expansion of the LDCT mass screening into low-risk subpopulation [29]. The NLST trial was conducted among high-risk individuals of old age and heavy smokers, and demonstrated positive results [4]. The NLST participants with a negative LDCT screen at baseline had a lower incidence of lung cancer and lung cancer-specific mortality compared to all participants [30]. An extrapolation of NLST trial in Germany found 20% lower relative risk of lung cancer death among heavy smokers aged 55-74 years [31]. In a LDCT screening cohort in western China similar to participants in the NLST trial, Lei, et al. found the number of risk factors of lung cancer was associated with increasing detection rate of asymtomatic lung cancer [32]. Providing a strict design and conduction, the benefits of LDCT screening in high-risk populations for lung cancer may overweigh those harms [33]. However, there is no high-quality evidence based on Chinese population to define the specific high-risk subpopulation as screening candidates. China is a large market of tobacco consumption, and the society appears the phenomenon of population aging. Therefore, regarding the balance of benefits and harms, the criteria for screening candidates in the NLST trial is referrable to the health care system in China.

Moreover, the DANTE trial (based on a community setting) and the MILD trial didn't find confirmative results to support the LDCT mass screening against none screening [34, 35]. In the aspect of a national plan, introducing some supplementary indicators for surveillance would be more cost-effective. In the practice, identificatoin of emphysema in LDCT scan is associated with an increased rate of lesion detection [36]. Namely, adding emphysema into the criteria for the more intensive LDCT surveillance may be an effecient approach to increasing detection rate of lung cancer. Pulmonary focal ground-glass opacities might frequently be identified by the LDCT screening, and partially associated with the high rate of false-positivity [37]. Jiang, et al. established a modified prognostic scoring system, which demonstrated a good performance in identifying benign and malignant opacities [37]. Development of a risk prediction model for lung cancer may discriminate high-risk from low-risk individuals based on the associations with demographic and environmental factors.[38]. Besides, a comprehensive lung cancer control plan in high risk areas should combine screening with tobacco control and health education, especially in population with low education level [39].

Therefore, recently, the lung cancer early detection and treatment expert group, appointed by the National Health and Family Planning Commission, launched a China national lung cancer screening guideline in the 2015, based on current available high-quality evidence and protocol of lung cancer screening program conducted in rural China [40]. Annual LDCT screening joint with health education assisting smoking cessation is recommended for high risk individuals aged 50-74 years who have at least a 20 pack-year smoking history and who currently smoke or have quit within the past five years [40]. This guideline aims to translate the advancement through LDCT mass screening in western nations into increasing proportion of stage I-II lung cancers in Chinese population, and consequently lead to better population survival outcome of lung cancer and more related life years saved.

Finally, there is a concern whether the relationship between the early detection of lung cancer and the survival improvement might be confounded by the lead-time bias or not [41]. As known, to estimate the confounder effect from the lead-time bias, the mortality rate of the disease need look at instead as a gold standard for measuring the early screening and treatment, but not the survival length. Namely, the events of lung cancer-related death during the long-term follow-up should be considered between early and locally advanced stages. Additionally, the most of the lung cancer-related death are resulted from postoperative recurrence or metastatasis. Therefore, a comparison of postoperative recurrence or metastatasis is also meaningful to test the lead-time bias. Torok JA, et al. found, among surgical patients with stage I-III non-small cell lung cancers, the earlier T stage or N stage diseases displayed a lower distal metastatasis risk [42]. Furthermore, Wang J, et al. evaluated the natural growth and disease progression of lung cancer through 18F-fluorodeoxyglucose PET/CT, and found the lung cancer appeared rapid tumor volume growth with the double time no more than half a year [43]. Actually, the lead time derived from lung cancer screening might be very limited. Therefore, we guess the the lead-time bias influences little in the lung cancer sceening associated improvement of the population survival.

In a short, by the epidemiological inference, the LDCT screening might be associated with increasing stage I lung cancer and therefore improving population survival outcome. Therefore, regarding the poor population survival outcome, it's necessary to establish nationwide organized screening and surveillance programmes based on LDCT among high-risk population to improve overall survival outcome of lung cancer in China. However, how to translate the experiences of lung cancer screening by LDCT from developed counties to China in a cost-effective manner needs to be further investigated.

## MATERIALS AND METHODS

The proportion of stage I lung cancer in LDCT screening population was retrieved from NLST (U.S.) and NELSON (Europe) trials [4, 5]. The general proportion of stage I lung cancer in China was retrieved from a rapid meta-analysis with a random effect model (Comprehensive Meta-Analysis 2.0; Biostat, Englewood NJ, USA). The China National Knowledge Infrastructure (CNKI) database was searched during 2012–2014. Only studies based on hospital-based cross-sectional population were included for meta-analysis. The lung cancer mortality and prevalence of China, U.S. and Europe was retrieved from Globocan 2012 fact sheet issued by International Agency for Research on Cancer [44]. Mortality-to-prevalence ratio (MPR) was applied to compare the population survival outcome of lung cancer [8, 45, 46]. The relative ratio (RR) between China and U.S. or Europe was calculated to estimate the differences of stage I disease proportion and population mortality. Two-sided p value less than 0.05 was considered as statistical significance.

### Data sharing

The reference list of included Chinese studies identified from the China National Knowledge Infrastructure (CNKI) database was unpublished. These extra data is available by emailing Dr. Xin-Zu Chen (chen_xz_wch_scu@126.com).
